# Genetic variation of FcγRIIa induces higher uptake of *Leishmania infantum* and modulates cytokine production by adherent mononuclear cells *in vitro*

**DOI:** 10.3389/fimmu.2024.1343602

**Published:** 2024-02-22

**Authors:** Jonatas da Silva Catarino, Rafael Faria de Oliveira, Marcos Vinicius Silva, Helioswilton Sales-Campos, Fernanda Bernadelli de Vito, Djalma Alexandre Alves da Silva, Lucila Langoni Naves, Carlo José Freire Oliveira, Denise Bertulucci Rocha Rodrigues, Virmondes Rodrigues

**Affiliations:** ^1^ Laboratory of Immunology, Institute of Natural and Biological Sciences, Federal University of Triângulo Mineiro, Uberaba, MG, Brazil; ^2^ Institute of Tropical Pathology and Public Health, Federal University of Goiás, Goiânia, GO, Brazil; ^3^ National Institute of Neuroimmuno Modulation, Rio de Janeiro, Brazil

**Keywords:** leishmaniasis, FcγRIIa (CD32a), polymorphism, AMCs, infection, phagocytosis

## Abstract

**Introduction:**

Single nucleotide variations (SNVs) are specific genetic variations that commonly occur in a population and often do not manifest phenotypically. However, depending on their location and the type of nucleotide exchanged, an SNV can alter or inhibit the function of the gene in which it occurs. Immunoglobulin G (IgG) receptor genes have exhibited several polymorphisms, including rs1801274, which is found in the FcgRIIa gene. The replacement of A with T results in a Histidine (H) to Arginine (R) substitution, altering the affinity of the IgG receptor for IgG subtypes and C-reactive protein (CRP). In this study, we analyzed rs1801274 and its functional implications concerning L. Infantum uptake and cytokine production.

**Methods:**

We genotyped 201 individuals from an endemic area for visceral leishmaniasis to assess the presence of rs1801274 using Taqman probes for a candidate gene study. Additionally, we included seventy individuals from a non-endemic area for a functional study. Subsequently, we isolated and cultivated one-week adherent mononuclear cells (AMCs) derived from the peripheral blood of participants residing in the non-endemic region in the presence of L. infantum promastigotes, with and without antigen-specific IgG and/or CRP. We analyzed the rate of phagocytosis and the production of nitric oxide (NO), tumor necrosis factor (TNF)-a, interleukin (IL)-10, IL-12 p70, IL-1b, IL- 6, and IL-8 in the culture supernatants.

**Results and discussion:**

In participants from the endemic region, the A/G (H/R isoform) heterozygous genotype was significantly associated with susceptibility to the disease. Furthermore, SNVs induced a change in the phagocytosis rate in an opsonin-dependent manner. Opsonization with IgG increased the production of IL-10, TNF-a, and IL-6 in AMCs with the H/R isoform, followed by a decrease in NO production. The results presented here suggest that the rs1801274 polymorphism is linked to a higher susceptibility to visceral leishmaniasis.

## 1 Introduction

Leishmaniasis is a group of diseases caused by protozoa belonging to the genus Leishmania (1, 2). It affects more than 12 million of people worldwide, with 92 countries or territories currently considered endemic for cutaneous leishmaniasis and 83 for visceral leishmaniasis, as reported by the World Health Organization in 2018 and Pan American Health Organization (3, 4).

The infectious process begins when female sandflies of the genus Phlebotomine bite a host and introduce infective forms known as metacyclic promastigotes. Phagocytic cells, including neutrophils, dendritic cells, and macrophages, are quickly attracted to the infection site and are capable to uptake the promastigotes. Inside of the macrophages phagocytic vacuoles, the parasite undergoes a transformation into amastigotes, which are tissue forms that replicate within macrophages. After a few replicative cycles, the parasite lyses the macrophages and proceeds to infect the next mononuclear cell (5). The interaction between these macrophages and the parasite ultimately determines the outcome of the infectious process.

The infection can be asymptomatic or manifest in various forms, the latter including cutaneous and visceral forms. The visceral form, if left untreated, can be fatal. The clinical manifestations of this disease depend on several factors, including the species of *Leishmania* causing the infection and the host’s immunological status, particularly in the early stages of the infection (6).

The clinical manifestations of visceral leishmaniasis (VL) generally include prolonged fever, hepatosplenomegaly, weight loss, pancytopenia, and hypergammaglobulinemia. These symptoms can progress to severe and often fatal complications such as hemorrhage (7).

The disease is characterized by the release of several proinflammatory cytokines, a phenomenon described as a cytokine storm (8, 9). Additionally, individuals with VL often exhibit an inability of peripheral blood mononuclear cells (PBMCs) to respond to stimulation with Leishmania antigen. However, this impairment tends to improve after treatment (10).

Phagocytic cells, such as neutrophils, dendritic cells and macrophages, are rapidly recruited to the site of infection shortly after parasite inoculation by the vector. However, the parasites are able to modulate various pathways of internalization used by these cells. For instance, infected neutrophils can be exploit by the parasite to enter macrophages without triggering its activation. This is achieved by inducing MCP-1 expression to attract macrophages and promoting neutrophil cell death. Macrophages, in turn, recognize the apoptotic bodies and engulf them passively, a mechanism referred to as the “Trojan horse” (11, 12). The parasite further modulates phagocytosis through the Mannose-Fucose Receptor (MR) and complement receptors (CR1 and CR3). Promastigotes predominantly uses CR3 to enter Macrophages, using GP63 to cleave C3b into iC3b, an inactive form that acts as an opsonin, binding to CR3. Notably, CR3 does not activate respiratory burst (13). The parasite can prevents phagosome-lysosome fusion, residing inside a modified phagolysosome to avoid degradation (14). This modulation creates a trade-off situation, wherein cytokine production, such as IL-2 and IL-10, is influenced while the microenvironment of infection is altered, ultimately enhancing the parasite’s chances of survival.

Receptors responsible for recognizing *Leishmania* can be categorized into at least four major groups among them: complement receptors (CR1 and CR3), MR, fibronectin receptors (FR), and immunoglobulin Fc receptors (FcR) (15). Interaction with some receptors, including FcγR, has been shown to modulate signaling pathways. Previous studies have demonstrated that the activation of stimulated murine macrophages in the presence of immunocomplexes (ICs) selectively decreases the production of interleukin (IL)-12 while increasing the synthesis and secretion of IL-10 (16, 17). Exploring this mechanism further, another study revealed that specific *Leishmania* ICs impair the response of Leishmania-infected macrophages primarily by increasing IL-10 production (18). Collectively, these effects contribute to the parasite’s survival strategy.

Studies have been conducted to identify the genetic factors responsible for increased susceptibility to infectious diseases in specific populations (19). Genetic mapping studies have pinpointed regions in the DNA associated with this heightened susceptibility, and some of these regions correspond to areas of the DNA responsible for encoding components of the immune system. This includes certain cytokines and immunological receptors like FcγRIIa (CD32a) (20). The Fc receptor family is expressed on various immune cells, including macrophages, dendritic cells, and other cells involved in the host-parasite interaction during visceral leishmaniasis. Notably, CD32a is primarily expressed on macrophages that have been differentiated *in vitro* (21).

CD32a is a low-affinity receptor that interacts with all four Immunoglobulin G (IgG) isoforms (IgG1, IgG2, IgG3, and IgG4) but with varying affinities. Notably, it is the exclusive receptor for IgG2. The recognition of these different IgG isoforms is strongly influenced by single nucleotide variations, primarily rs1801274. In rs1801274, a substitution occurs at position 131 in the protein, where Histidine (H) is exchanged with Arginine (R), leading to alterations in the receptor’s ability to bind to IgG2/IgG3 (22). The H131 receptor effectively binds to IgG molecules, whereas the R131 variant loses its affinity for these IgGs and exhibits an increased ability to interact with other opsonins, particularly C-reactive protein (CRP), along with pentraxins (23, 24).

In this study, we examined the H/R 131 polymorphism in the CD32a molecule as a potential biomarker for increased susceptibility to *L. infantum* infection, a susceptibility previously observed in individuals residing in endemic areas. Additionally, we investigated whether this polymorphism has an impact on susceptibility or resistance to *L. infantum* infection *in vitro*. Our findings revealed that the proportion of individuals expressing the H/R isoform was higher among VL-positive participants living in the endemic area. Furthermore, when we infected AMCs derived from individuals with the H/R isoform *in vitro*, we observed an immunological profile similar to that observed in individuals with active VL.

## 2 Methods

### 2.1 Participants

Two hundred and one individuals from an endemic area (Paracatu-MG Brazil) were recruited, and the frequency of the polymorphism was surveyed among them. Of these individuals, seventy-five were positive for VL, while one hundred and twenty-six were used as negative controls for VL; however, they all shared the same environment. For the *in vitro* functional analysis, seventy individuals from the non-endemic area, who tested negative for VL and had no clinical signs and symptoms of other comorbidities or recent infections, were selected. All samples were genotyped using the qPCR technique for the SNV rs1801274. After the genotyping, ten individuals from each genotype were selected for *in vitro* analyses. All individuals agreed to participate in this study by signing a consent form. This study was approved by the ethics committee of the Federal University of Triângulo Mineiro.

### 2.2 DNA purification and allelic discrimination using qPCR

The SNV at position 497, A/G (H/R) (missense His131Arg), of the FcγRIIa (rs1801274) was analyzed in this study. For this, 4–8 mL of peripheral blood was collected in vacuum collection tubes (BD-BRASIL) containing EDTA and taken to the Immunology Laboratory of the Federal University of Triângulo Mineiro; the DNA extraction process was performed using the Wizard^®^ Genomic DNA Purification Kit Technical Manual according to the manufacturer’s instructions. The allelic discrimination of the polymorphism was performed using the Applied Biosystems-TaqMan^®^ SNP Genotyping Assays system.

### 2.3 Culturing the *parasite*

The IOC/L2906 (MHOM/BR/2002/LPC-RPV) strain of *L.(L.) infantum* was cultivated in Schneider’s medium supplemented with 20% inactivated fetal bovine serum, 1% sodium pyruvate, 1% L-glutamine, calcium chloride 750 mg/l, and 40 g/ml gentamicin. The complete medium was sterilized by membrane filtration (0.22 μm), and the parasites were cultured in plastic flasks at 26°C.

### 2.4 Isolation of one-week AMCs (macrophages) from peripheral blood

The mononuclear cells in the peripheral blood samples collected were separated by density gradient using Ficoll-Hypaque (Pharmacia-Uppsala, Sweden). The number of cells obtained was determined by counting in a Neubauer’s chamber, and the volume of the medium used was adjusted to obtain 2x10^6^ cells/ml. The cells were cultured in complete RPMI containing 50 mM Hepes (GIBCO-USA), 5% inactivated fetal bovine serum, 2 mM L-glutamine (GIBCO-USA), and 40µg/ml gentamicin (ARISTON-BRASIL). After 4 hours, the cells were washed to remove those that were non-adherent, and then the AMCs cultured for 7 days to allow the differentiation of cells morphologic and transcriptionally similar to macrophages (25, 26).

### 2.5 Purification of CRP

Serum samples obtained from individuals with high concentrations of CRP were used to prepare a pool which had a concentration of 254.2 mg/dL. The pool was incubated with a solution containing anti-CRP antibodies in latex particles for 1 hour under continuous shaking to allow for an interaction between the antibodies and CRP. Next, the solution was centrifuged, and the pellet, which contained the complex formed by the human CRP and latex particles, was resuspended in Tris-HCl solution at pH 5 and incubated for 15 minutes to promote the breakdown of this complex. At the end of the incubation, the solution was filtered using 0.22μm-syringe filters. The filtrate had its pH adjusted to 7 after the CRP concentration was measured using a diagnostic kit that operates with the turbidimetry methodology, where only PCR is recognized in its pentameric structural conformation. The solution was stored at 4°C until the date of use.

### 2.6 Quantification of IgG isotype and purification of specific parasite immunoglobulins

Serum from participants with acute and chronic VL was used to prepare a pool of *L. infantum-*specific IgG. The participants were classified as acute and chronic following the clinical and laboratorial criteria: patients in the acute phase were symptomatic and present fever, weight loss, fatigue, enlarged spleen and liver (hepatosplenomegaly), and anemia and have positive serological test, chronic cases were tested positive before, received the treatment and show clinical cure, however, were still with the positive serological test (27). Prior to this, the total amount of IgG and the amount of each subtype were quantified in the pool by ELISA. Then, the pool was incubated with protein A (ProtA), which is associated with a polymer, for 1 hour to promote the interaction between ProtA and IgG. Next, the ProtA–IgG complex was centrifuged, and the pellet was resuspended in Tris-HCl (pH 3.5) to reverse the association of the specific ProtA–IgG complex. Then, the solution was incubated for 15 min and centrifuged. The supernatant, which only contained IgG isoforms IgG1, IgG 2, and IgG4 since protA does not bind to IgG3, was collected and transferred to a new tube, and the pH was adjusted to 7. The solution was stored at 4°C until the date of use.

### 2.7 Fixation and labeling of Leishmania infantum promastigotes

At the end of the exponential phase, the parasites were collected from cell culture flasks, centrifuged, and washed with sterile saline three times. Then, they were fixed in 4% paraformaldehyde and stored at 4°C. The fixed *L. infantum* suspension was centrifuged at 800xG for 30 minutes at 4°C, washed, and resuspended in carbonate buffer 0.05 M/0.15 M of NaCl containing 100 μg/ml of fluorescein isothiocyanate (FITC) before being incubated overnight at 4°C. After incubation, the sample was washed by centrifugation, resuspended in 1X PBS pH 7, and mounted on a slide (with a coverslip) to observe the labeling.

### 2.8 Opsonization of promastigote forms

For the study on opsonization, promastigote forms of *L. infantum* were fixed in 4% paraformaldehyde for 15 minutes at 4°C. Then, they were washed 3 times by centrifugation at 800xg for 20 min and labeled with FITC as described below.

**Opsonization Protocol 1:** *L. infantum* promastigotes, which had been prepared as described above, were incubated with 5 μg/ml of purified CRP in a culture medium for 1 hour at room temperature (RT). Afterward, they were washed 3 times with PBS 1X (to remove proteins that did not bind to the parasites’ surface) and resuspended in a culture medium without FBS.
**Opsonization Protocol 2:** *L. infantum* promastigotes, which had been prepared as described above, were incubated with specific antigen-specific IgGs at a dilution of 1: 800 (sub-agglutination concentration), pre-standardized dilution, in a culture medium for 1 hour at RT. Afterward, they were washed 3 times with PBS 1X (to remove the immunoglobulins that did not bind to the parasites’ surface) and resuspended in a culture medium without FBS.
**Opsonization Protocol 3:** *L. infantum* promastigotes, which had been prepared as described above, were incubated with CRP and antigen-specific IgG at a concentration of 5μg/ml and dilution of 1:800, respectively, in a culture medium for 1 hour at RT. After incubation, they were washed 3 times with PBS 1X (to remove the CRP and immunoglobulins that did not bind to the parasite surface) and resuspended in a culture medium without FBS.

### 2.9 *In vitro* infection

After isolating the PBMCs by density gradient and differentiating the AMCs by cell adhesion, on the sixth day after cell adhesion in the 48-well culture plates (SARTEDT-USA), the morphology of the remaining adherent cells was examined; the cells were bigger and showed extensions, characteristics of human AMCs derived from peripheral blood. Then, the promastigotes of *L. infantum* were added to the wells under pre-established opsonization conditions, in the absence of LPS and the presence of 1μg of LPS.

All experiments were performed in triplicate.

### 2.10 Analysis of phagocytosis rate (PR)

PR analysis was performed by counting the number of AMCs that exhibited diffuse green fluorescence in their cytoplasm after *in vitro* infection, a characteristic they assumed after internalizing FITC-labeled *L. infantum* promastigotes. Five different fields were counted from the culture well (up to 400 cells) to determine the percentage of cells that were involved in phagocytosis (28). The culture wells were previously analyzed to ensure that phagocytosis occurred homogeneously in them.

### 2.11 Cytokine quantification

The production of IL-10, IL-6, tumor necrosis factor (TNF)-α, IL-12, IL-1β, and IL-8 was analyzed simultaneously in the culture supernatants of AMCs using the Cytometric Bead Array (CBA) Human Inflammatory Cytokine Kit (BD Biosciences), according to the manufacturer’s instructions. The samples and recombinant cytokines were incubated with microspheres of different fluorescence intensities which had been conjugated with capture antibodies specific for each cytokine. Then, PE-conjugated antibodies specific for each cytokine were added. After incubation, the microspheres were washed with the corresponding solutions and analyzed on a FACSCalibur cytometer (BD Biosciences), using the CellQuest software (BD Biosciences). The results were analyzed using the FCAP Array 2.0 software (Soft Flow, Pécs, Hungary).

### 2.12 Nitric oxide (NO) quantification

The Griess reaction was performed to evaluate the production of nitrite in the AMCs culture supernatant as an indirect predictor of NO production. Fifty microliters of culture supernatant was incubated in 96 well plates (plates description), along with 50 microliters of revelation solution which was composed of Solutions A (Sulfanilamide 5% H_3_PO_4_) and B (0.1% NEED diluted 1:1). The reaction was read in a spectrophotometer, and the absorbance was measured in the 554 nm wavelength. The results were quantified using the standard curve of NaNO_2_ and expressed in uM.

### 2.13 Data analysis and statistics

For all variables, normal distribution and homogeneous variance were tested. The *D’Agostino-Pearson* test was used to assess normality for all variables. Multiple comparisons relating to the medians of values for more than two groups were made using the Kruskal-Wallis nonparametric test, followed by Dunn’s test. The observed differences were considered significant when *p <*0.05 (5%). Statistical analysis was performed using the GraphPad Prism software (GraphPad Software 8.0, La Jolla, CA, USA).

## 3 Results

### 3.1 The proportion of individuals with the H/R genotype was higher within the affected population

FcγRs are receptors which are associated with the internalization of Leishmania parasites during infection; they play a key role in removing circulating immune complexes. To demonstrate whether the A/G131 polymorphism is associated with infection susceptibility, we analyzed the frequency of polymorphism in a group of 75 individuals with VL and 126 healthy controls from the same area (endemic area). Interestingly, the proportion of heterozygous individuals was higher (60%) among the affected population compared to the other genotypes (**Table 1**).

**Table 1:** Genotypic distribution and allelic frequencies in individuals with Leishmaniasis and in controls for the H/R131 polymorphism in the *FcγRIIa* gene in an endemic zone.

Polymorphism		Active	Controls	Total	χ2, p
	**AA(H/H)**	7 (9.3%)	21 (16.7%)	28 (13.9%)	
	**AG(H/R)**	45 (60.0%)	53 (42.1%)	98 (48.8%)	0.0421*
**FcγRIIa (His131Arg) rs1801274**	**GG(R/R)**	23 (30.7%)	52 (41.3%)	75 (37.3%)	
	**A**	39.3%	37.7%	38.3%	0.3880
	**G**	60.7%	62.3%	61.7%	

*ORAG x GG=1,9 – IC=1,021 – 3,610; ORAG x AA=2,6 – IC=0,991 – 6,543.

The bold values denote statistical significance.

### 3.2 Frequency of polymorphism in the population studied *in vitro*

To analyze the putative mechanism underlying the higher frequency of heterozygous individuals within the affected population, a group of 70 individuals without VL was recruited, and blood samples were collected and genotyped to determine the frequency of the polymorphism. Again, the frequency of heterozygous individuals was higher compared to the other genotypes, three samples were removed of the study due technical issue during the genotyping process. (**Table 2**).

**Table 2:** Genotypic distribution and allelic frequencies for the H/R131 polymorphism of the *FcγRIIa* gene, alleles in individuals from the functional study.

Polymorphism	Genotype/isoform	Individuals
	**AA(H/H)**	12 (17.9%)
	**AG(H/R)**	35 (52.2%)
	**GG(R/R)**	20 (29.9%)
FcγRIIa (His131Arg) rs1801274		
	**A** **G**	44.03% 55.97%

### 3.3 The H/R isoforms influence the phagocytosis profile of AMCs challenged with L. infantum

**Figure 1:**
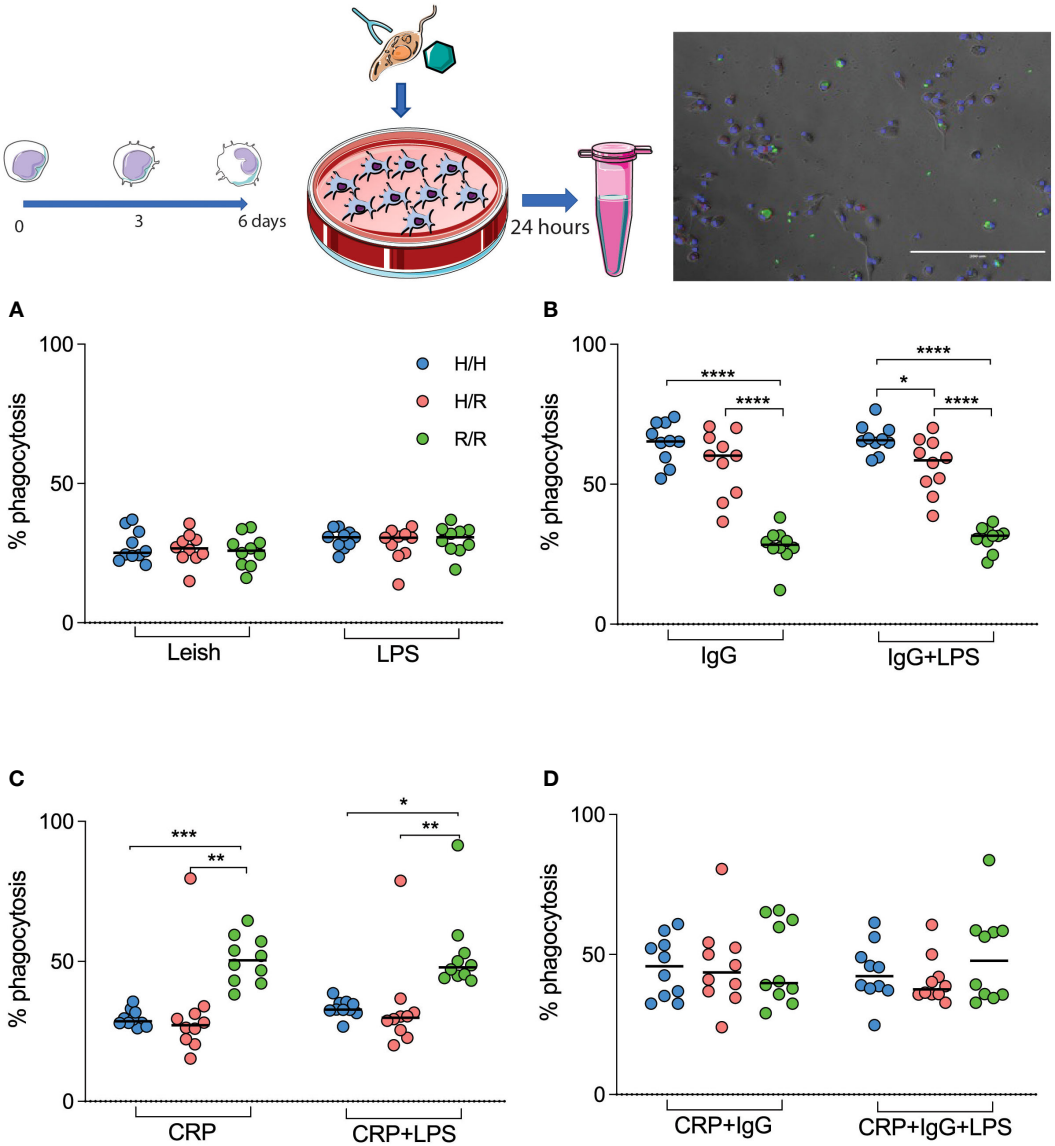
H/R131 AMCs differentially phagocytose opsonized *L. infantum*. AMCs isolated from peripheral blood are infected with *L. infantum* promastigotes **(A)**, promastigotes with specific pathogenic IgG **(B)**, promastigotes covered with CRP **(C)**, and promastigotes coated with both opsonins **(D)**. Results are expressed (median, quartile, and all points) as percentages of infected cells in one hundred cells. *p <0.05 **(p < 0.003), ***(p < 0.001) and ****(p < 0.0001).

To analyze the PR of AMCs with different isoforms of the A/G131 gene, we challenged them with *L. infantum* coated with human IgG-specific antigens and CRP since the polymorphism alters the receptor’s ability to bind to these two molecules which classically work as opsonins. The differences in isoforms did not influence the PR when AMCs were exposed to non-opsonized promastigotes (**Figure 1A**). Interestingly, when parasites were opsonized with human IgG, AMCs with the H/H and H/R isoforms showed a significant increase in PR compared to those with the R/R genotype (**Figure 1B**). However, when the parasites were opsonized with CRP, this phenomenon was reversed, and AMCs with the R/R isoform demonstrated a significant increase in PR compared to those with the H/H and H/R isoforms (**Figure 1C**). When double opsonization was performed, there was no difference in PR among AMCs with the three isoforms, although the rate of phagocytosis increased for all groups (**Figure 1D**). Therefore, PR seemed to be influenced by the polymorphism, and this phenomenon was dependent on the opsonin covering the parasite. Interestingly, AMCs with the H/H and H/R isoforms demonstrated similar phagocytosis profiles.

### 3.4 The H/R131 polymorphism promotes NO production in the AMCs of R/R individuals

NO is one of the molecules produced during the process of AMCs (macrophages) activation downstream of the activation of NADPH oxidase and is responsible for the fight against intracellular bacteria and protozoa. Thus, it is of great importance to analyze if the H/R131 polymorphism may impact the production of this molecule once PR is altered at opsonin and genotyped dependent.

**Figure 2:**
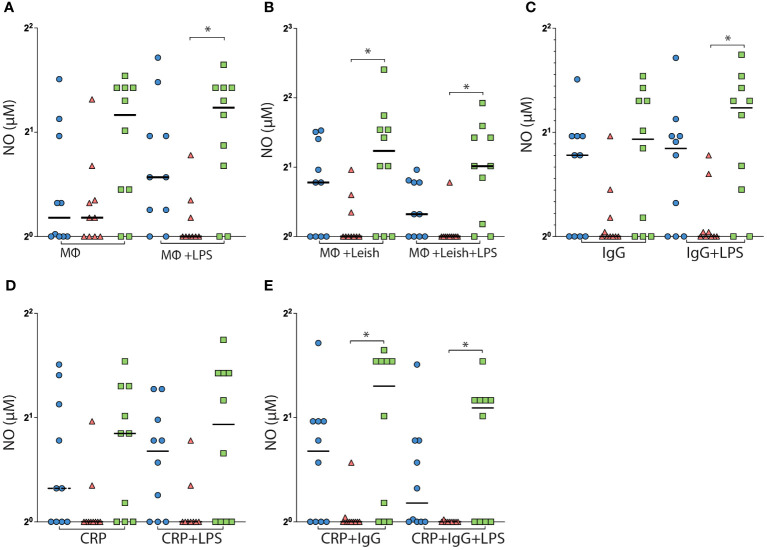
H/R131 AMCs affect NO production. AMCs isolated from peripheral blood were infected with *L. infantum* promastigotes and analyzed for the production of NO. AMCs not infected only in the culture medium **(A)**, AMCs infected with the promastigotes of naked *L. infantum* not covered with opsonins **(B)**, AMCs infected with *L. infantum* coated with IgG- pathogen-specific **(C)**, AMCs infected with *L. infantum* coated with CRP **(D)**, and AMCs infected with *L. infantum* coated with IgG- pathogen-specific and CRP **(E)**. Results are expressed median and all points.

Comparing AMCs with the R/R isoform with those from heterozygous individuals, the former was observed to induce greater NO production under the following conditions: infected AMCs, AMCs infected with naked Leishmania, AMCs infected with IgG coated-Leishmania, and double-coated AMCs (**Figure 2**). Regarding the role of CRP in this scenario, no differences were observed among the groups (**Figure 2E**).

### 3.5 H/R131 influences the production of key cytokines in the innate immune response against L. infantum

An individual’s susceptibility to infectious diseases such as VL is determined by different factors including environmental factors, the species of the microorganism, and the host’s genetic background. The host’s genetic background, in many cases, has a direct influence on the immune repertoire that will be set up in the presence of infection. To address the impact of the H/R131 polymorphism on the activation of AMCs, we measured the production of cytokines in the supernatant culture of AMCs expressing the different isoforms of this receptor in presence of the parasite coated with opsonin molecules, as previous described in the Methods section.

**Figure 3:**
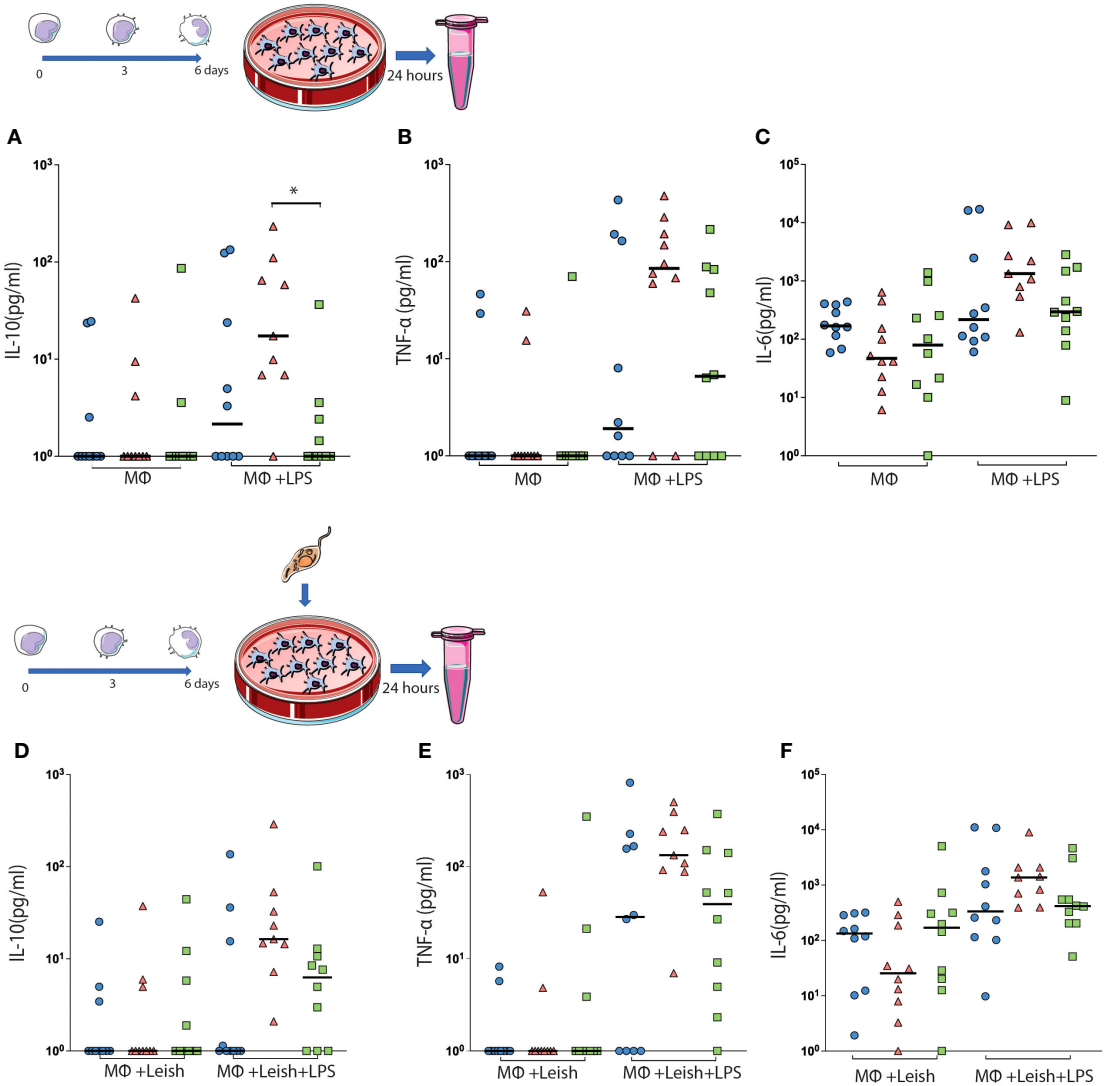
The concentrations of IL-10 **(A, D)**, TNF-a **(B, E)** and IL-6 **(C, F)** were detected in the supernatant of the cell culture. AMCs were isolated from peripheral blood and analyzed to produce oxide key cytokines for the polarization of these cells. The concentrations of IL-10 **(A)**, TNF-α **(B)**, IL-6 **(C)**, and IL-8 **(D)** were detected in the supernatant of the cell culture. The results are expressed (median and all points) in picogram per milliliter (pg/ml/g). *p < 0.05.

Non-infected AMCs expressing the H/R isoform showed a significant increase in the production of IL-10 compared to those expressing the R/R isoform (**Figure 3A**). However, no differences in the production of other cytokines were observed in this condition. When the AMCs were infected with naked *Leishmania*, no differences in the production of cytokines and chemokines were observed between the groups (**Figures 3D–F**).

**Figure 4:**
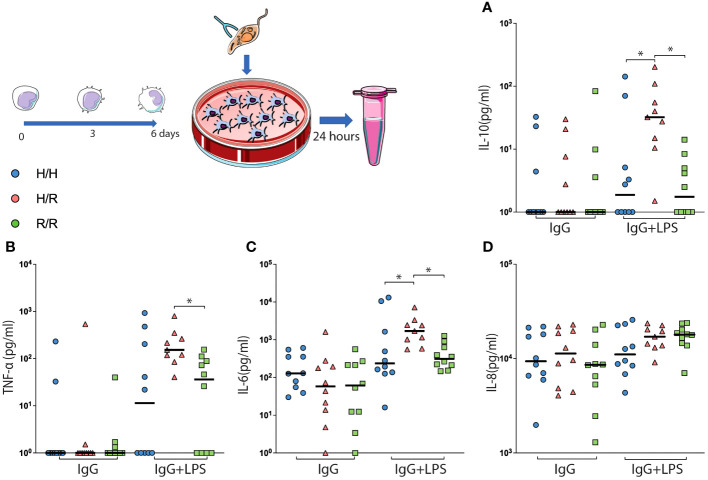
AMCs with the H/R isoform showing a cytokine storm-like phenomenon after infection with leishmania parasite coated with IgG. AMCs were isolated from peripheral blood, infected with *L. infantum* coated with pathogen-specific IgG, and analyzed for the production of oxide key cytokines for the polarization of these cells. The concentrations of IL-10 **(A)**, TNF-α **(B)**, IL-6 **(C)**, and IL-8 **(D)** were detected in the supernatant of the cell culture. The results are expressed as picograms per milliliter (pg/ml/g) in a bar plot with means and SEM and all the points. *p < 0.05.

We then analyzed the production of pro- and anti-inflammatory cytokines—TNF-α, IL-6, CXCL8 (IL-8), IL-12, IL-1β, and IL-10—in AMCs with different *L. infantum*-challenged isoforms to determine whether the activation profile of these cells is modulated by specific IgG–pathogen ICs. We observed that AMCs expressing the H/R isoform produced higher levels of IL-10, TNF-α, and IL-6 compared with those expressing the R/R isoform (**Figures 4A–C**) and higher levels of IL-10 and IL-6 (**Figures 4A, C**) compared to those expressing the H/H isoform. There were no differences in the production of chemokines and cytokines between the groups.

When the AMCs with different isoforms of *L. infantum* were challenged with either CRP or IgG+CRP (**Supplementary Table 1**), no differences in the production of cytokines and chemokines were detected between them. Furthermore, the production of IL-1β and IL-12 was not detected (Data not shown).

Taken together, these results suggest that AMCs with the H/R isoform exhibit increased production of IL-10, TNF-α, and IL-6, in the presence of IgG, which is associated with a lower activation and production capacity of parasiticide molecules such as NO.

## 4 Discussion

The mechanism underlying increased susceptibility to infectious diseases, such as visceral leishmaniasis (VL), is intricate and involves numerous factors, including age, nutritional status, and the genetic characteristics of the host. Both host and pathogen genetics significantly influence the infection’s outcome (19). In this study, we suggest that the polymorphism in the FcγRIIa receptor may play a role in determining an individual’s susceptibility or resistance profile to *L. infantum* infection. Differences in the phagocytosis profile among genotypes, coupled with an observed increase in IL-10 production and a decrease in nitric oxide (NO) production in AMCs derived from individuals with the H/R variant, may contribute to a heightened susceptibility in individuals carrying this single nucleotide variation (SNV) when exposed to *L. infantum* infection.

The ability of FcγRIIa to bind to immunoglobulins is directly affected by the H/R131 polymorphism. This single nucleotide variation (SNV) leads to an exchange of Histidine (H) for Arginine (R), resulting in altered affinity of the receptor for IgG molecules of different isoforms (29). It has been demonstrated that the same polymorphism influences the affinity of FcγRIIa for CRP. Furthermore, the binding of the R131 isoform with CRP triggers a temporary increase in intracellular calcium ion (Ca2+) levels, indicating a functional interaction and the capacity to activate downstream elements in this intracellular network (23). This could potentially be the mechanism behind the observed alteration in the phagocytosis capacity of AMCs in our study, as changes in the cytoskeleton necessary for phagocytosis rely on fluctuations in intracellular Ca2+ (30), and fixed parasites was used to eliminate the potential influence of a live organisms. The cellular uptake of pathogens coated with antibodies and other opsonins initiates intracellular signaling pathways that significantly influence the activation and polarization of AMCs. These pathways also affect their ability to eliminate pathogens and modulate the activation of adaptive immunity (21). Therefore, it is imperative to delve into the molecular mechanisms that underlie the alteration in phagocytosis capacity induced by this polymorphism.

Genetic studies in other complex diseases, such as malaria, have indicated that the CD32/FcγRIIa family of immunoglobulin receptors plays a significant role in disease outcomes. The polymorphism resulting in homozygosity for the R131 variant leads to reduced binding ability to IgG2, and individuals with this polymorphism are associated with a lower risk of developing malaria compared to those with the H/R heterozygous variant (31). In a different study it was identified an increased risk of infection with *P.falciparum* of infants carrying the variant H/R (32).

Our study has revealed that individuals with the H/R131 isoform exhibit higher production of IL-10, TNF-α, and IL-6, along with lower production of nitric oxide (NO) when compared to individuals with other isoforms. Additionally, we observed a higher prevalence of the H/R131 isoform among individuals with active disease residing in the endemic area. As previously mentioned, the polymorphisms strongly affect the receptor’s ability to bind to IgG2, which is the primary subclass present in the sample pool used in our experiments (**Supplementary Figure 1**). This suggests that the observed phenotype may be influenced by IgG2 binding to CD32. However, the significance of other subclasses in this context remains uncertain, and further studies are needed to elucidate the roles of various isoforms.

Studies in mice with depletion of the J locus of the immunoglobulin heavy chain have revealed that the absence of this locus leads to improved control of infection. This control is characterized by reduced lesion size, decreased parasite burden, and a significant reduction in IL-10 production, along with an increase in IL-12 production (18). Additionally, research has shown that animals lacking the ability to produce the common γ chain of the Fc receptor family are resistant to infection with *L. mexicana* amastigotes (33).

In human macrophages, when stimulated through FcγRs and LPS, there is a decrease in the production of inflammatory cytokines, primarily due to the autocrine action of IL-10. The synergistic action of these two receptors amplifies IL-10 production, leading to the rapid suppression of cellular activation (34). Furthermore, a polymorphism in the region encoding IL-10 has been associated with increased IL-10 production and susceptibility to infection by *L. braziliensis* in humans (35).

IL-6 is commonly recognized as a pro-inflammatoy cytokine that is produced in response to various inflammatory stimuli. However, in certain models, there has been a suggestion of a regulatory role for this cytokine (36). In an *in vitro* infection model with *T. gondii*, pre-treatment of macrophages with IL-6 has been observed to result in increased parasite multiplication within the parasitophagous vacuoles (37). Additionally, it has been proposed that IL-6 induces B cell proliferation through a receptor formed by gp86 and CD130, subsequently activating STAT1 and STAT3 (38). More recent research has highlighted IL-6 as an inducer of IL-21-producing CD8+ helper-like T cells. IL-6-activated TCD8+ helper-like T cells, similar to T CD4+ follicular cells, have been found to promote changes in antibody isotype and a significant increase in IgG1 production in B cells (39). It has been suggested that IL-6 may be associated with the activation and proliferation of plasma cells, contributing to the high production of immunoglobulins in individuals with active visceral leishmaniasis (VL). This phenomenon is associated with clinical worsening in VL cases.

It has also been demonstrated that TNF-α is elevated in the serum of individuals with active visceral leishmaniasis (VL), often in conjunction with high levels of IFN-γ. These elevated levels of TNF-α and IFN-γ may contribute to the activation of macrophages and the elimination of the parasite (40). However, it’s important to note that the systemic elevation of these cytokines in the bloodstream may not necessarily reflect their levels at the actual infection site (8). On the contrary, the presence of high levels of TNF-α in the serum of patients can be associated with complications in the clinical state due to its potentially harmful systemic effects (41).

Macrophages are the primary host cells involved in the immune response against parasites belonging to the *Leishmania* genus, and they play a crucial role in eradicating parasites when appropriately activated. The respiratory burst, which leads to the production of O2- and NO, is one of the primary mechanisms through which macrophages control infection. However, the precise role of NO in this context remains not fully understood. Studies have yielded somewhat conflicting results regarding the role of NO. For example, inhibiting NO production by blocking NADPH in monocytes/AMCs stimulated by *L. braziliensis* and IFN-γ did not lead to significant differences in the cells’ ability to eliminate internalized parasites (42). Conversely, another study involving iNOS KO (inducible nitric oxide synthase knockout) animals and IFN-γ KO animals indicated that lesions initially appeared in IFN-γ KO animals, suggesting that NO does indeed play an essential role in controlling parasitic growth (43). Therefore, while NO is associated with parasite control, it is also produced in response to infection and may not always contribute significantly to parasite eradication. Instead, in high concentrations, NO can potentially lead to tissue damage. In cases where anti-inflammatory cytokines like IL-10 are produced, they may be responsible for dampening the immune response, resulting in an inability to control parasitic growth and the progression of infection.

In summary, the results of our study, both from the genetic and functional aspects, suggest that heterozygosity for the FcγRIIa polymorphism is linked to an increased susceptibility to *L. infantum* infection. One possible explanation for this observation could be related to the receptor’s activation mechanism, which requires immune complexes (ICs) formed by a substantial number of IgG molecules rather than monomers (21). It has been observed that macrophages stimulated with high IC density exhibit reduced IL-12 production and increased IL-10 levels (44). Furthermore, the genetic inheritance pattern of FcγRIIa has been shown to promote the co-expression of high and low IgG receptors (45). This suggests that individuals expressing H/R isoforms might require a lower concentration of ICs to saturate the receptors (especially if they also bind to CRP), leading to alterations in cytokine production. This phenomenon may not have been observed in participants with the H/H isoforms. However, the exact mechanism underlying this phenomenon still requires further investigation. Overall, our data indicate that the H/R131 polymorphism in heterozygosis is associated with an increased susceptibility to visceral leishmaniasis (VL) in individuals residing in endemic regions. Furthermore, it disrupts the delicate balance necessary for infection resolution by modulating key cytokines responsible for infection control and significantly affecting parasite phagocytosis. Therefore, this receptor warrants further exploration as a potential target for the development of new treatment strategies. Additional studies with larger participant cohorts are needed to elucidate possible downstream signaling pathways that lead to the activation of different receptor variants.

## Data availability statement

The original contributions presented in the study are included in the article/**Supplementary Material**. Further inquiries can be directed to the corresponding author.

## Ethics statement

The studies involving humans were approved by 1.846.584 CEP/Universidade Federal do Triangulo Mineiro. The studies were conducted in accordance with the local legislation and institutional requirements. The participants provided their written informed consent to participate in this study.

## Author contributions

JC: Formal Analysis, Investigation, Methodology, Writing – original draft. RO: Investigation, Methodology, Writing – original draft, Data curation. MS: Formal Analysis, Investigation, Writing – original draft. HS-C: Investigation, Writing – original draft. DS: Data curation, Investigation, Writing – original draft. LN: Investigation, Writing – original draft. FB: Formal Analysis, Investigation, Writing – original draft. CO: Formal Analysis, Writing – review & editing. DR: Formal Analysis, Methodology, Writing – review & editing. VR: Conceptualization, Funding acquisition, Project administration, Supervision, Validation, Writing – review & editing, Resources.
