# Supplementary figures and images for: Genetic variation of FcγRIIa induces higher uptake of Leishmania infantum and modulates cytokine production by adherent mononuclear cells in vitro
Source: Front Immunol. 2024 Feb 22;15:1343602. doi: 10.3389/fimmu.2024.1343602 (PMC10917923; doi:10.3389/fimmu.2024.1343602)

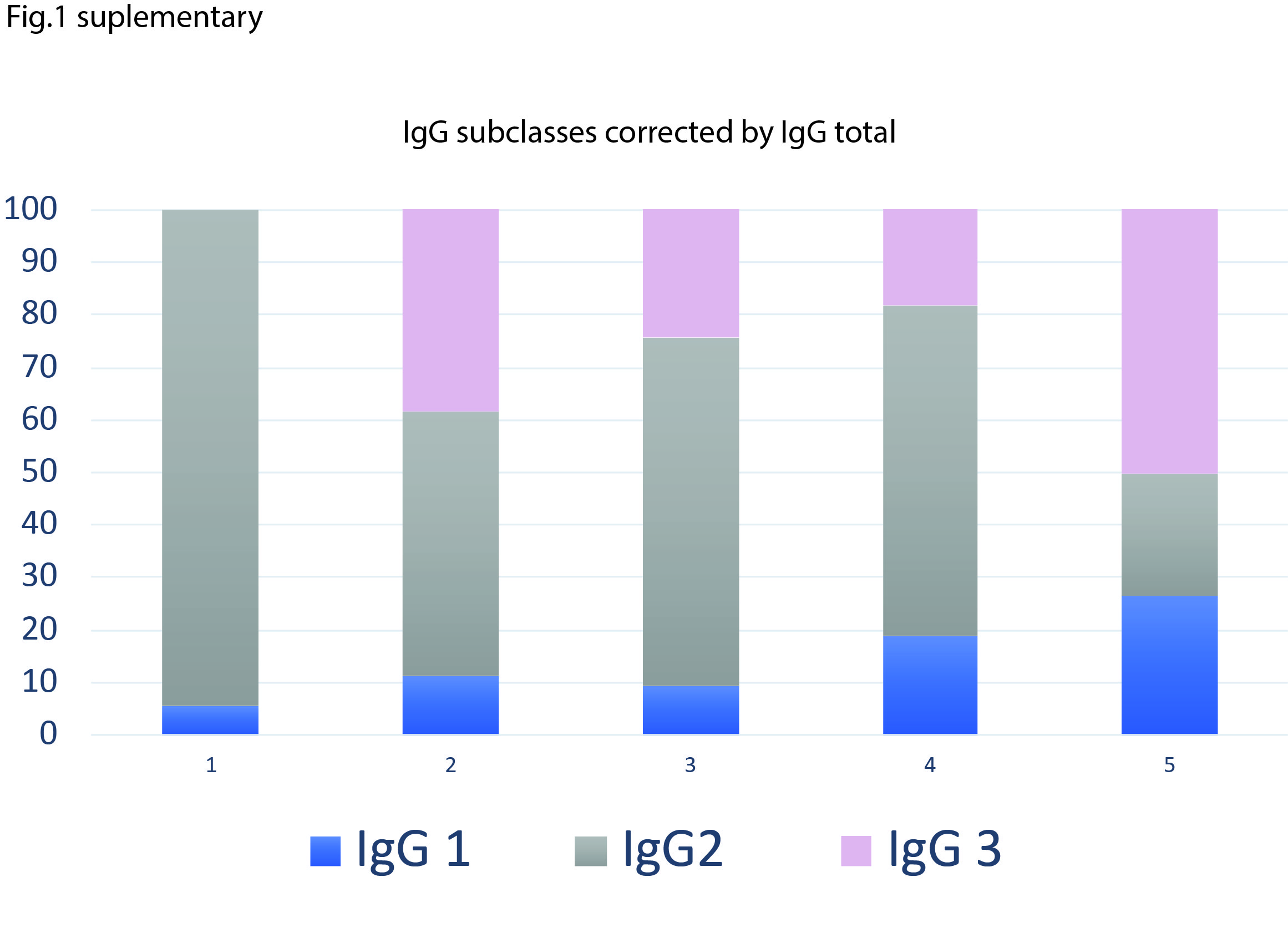

Supplement: Supplementary file 1 [file Image_1.jpeg]

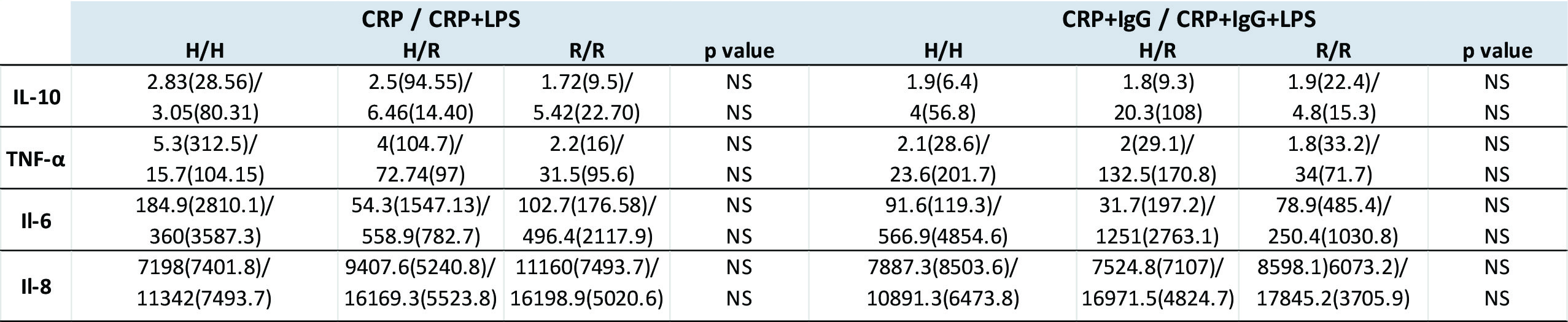

Supplement: Supplementary file 2 [file Image_2.jpeg]
